# Integrated Genomic Characterization of Aggressive Multiple Myeloma With 1q21 Gain Reveals IL6R‐Associated Chromatin Remodeling: A Case Report

**DOI:** 10.1002/cnr2.70744

**Published:** 2026-11-14

**Authors:** Haibo Liu, Mengshi Chen, Yue Wang, Ming Chen, Dong He, Yanju Li, Kaiji Zhang

**Affiliations:** ^1^ Chengdu University of Traditional Chinese Medicine Chengdu Sichuan China; ^2^ The First People's Hospital of Chengdu City Chengdu Sichuan China; ^3^ The Affiliated Hospital of Guizhou Medical University Guiyang Guizhou China

**Keywords:** 1q21 gain, chromatin remodeling, Hi‐C, IL6R, multiple myeloma

## Abstract

**Background:**

Chromosome 1q21 gain is a frequent secondary abnormality in multiple myeloma, yet its clinical impact depends heavily on coexisting genomic lesions. The regulatory consequences of this abnormality at the three‐dimensional chromatin level remain poorly understood.

**Case:**

An 80‐year‐old man with newly diagnosed multiple myeloma showed rapid disease progression following bortezomib–dexamethasone induction. He presented with severe cytopenias, marked hyperglobulinemia, extensive bone marrow infiltration, and rib destruction. Cytogenetic analysis identified 1q21 gain, del(13q), and a complex near‐triploid karyotype. Whole‐genome sequencing confirmed a broad copy number gain across 1q21 and copy number loss on 13q. Comparative transcriptomic profiling showed increased expression of several genes within or linked to the gained 1q21 region, with interleukin‐6 receptor (IL6R) showing the largest increase. Hi‐C analysis revealed local chromatin reorganization at the IL6R locus, including altered topologically associating domain organization, expansion of the local domain boundary, incorporation of distal enhancers, and increased enhancer‐promoter contacts. The patient showed no meaningful response to induction therapy and died within 1 month after the second cycle.

**Conclusion:**

This case suggests that 1q21 gain may be associated with an aggressive clinical course when it co‐occurs with del(13q) and a complex karyotype. Integrated genomic findings further suggest that IL6R‐centered chromatin remodeling may contribute to the biology of a subset of 1q21‐positive multiple myeloma.

**Abbreviations**
+1q/+1q21: gain/amplification of chromosome 1q/1q21
BM: bone marrow
bp: base pair
Chr: chromosome
CNV: copy number variation
DEG: differentially expressed gene
del(13q): deletion of the long arm of chromosome 13
DS: Durie–Salmon staging system
FC: fold change
FISH: fluorescence in situ hybridization
GO: Gene Ontology
Hi‐C: high‐throughput chromosome conformation capture
IgG: immunoglobulin G
IL‐6: interleukin‐6
IL6R: interleukin‐6 receptor
IMiD: immunomodulatory imide drug
IR: insulation score/insulation ratio
ISS: International Staging System
kb: kilobase
KEGG: Kyoto Encyclopedia of Genes and Genomes
Mb: megabase
MM: multiple myeloma
PI3K–AKT: phosphoinositide 3‐kinase–protein kinase B
TAD: topologically associating domain
TSS: transcription start site
WGS: whole‐genome sequencing

## 1 Introduction

Multiple myeloma (MM) is a clonal plasma‐cell malignancy with marked biological and clinical heterogeneity [1]. Contemporary treatment incorporating proteasome inhibitors, immunomodulatory drugs, monoclonal antibodies, cellular therapies, and transplantation has substantially changed the management of MM [2, 3]. Patients with biologically high‐risk disease, however, may still experience rapid progression, early relapse, and short survival despite modern therapy [3]. Defining the genomic features that distinguish these aggressive cases remains important for risk assessment and for understanding the biology underlying treatment failure.

Gain or amplification of chromosome 1q21 is among the most frequent secondary genomic abnormalities in MM. Evidence from randomized clinical trials has confirmed the adverse clinical relevance of chromosome 1q alterations [4]. Clinical and biological studies have also established 1q21 as a heterogeneous lesion whose significance varies across disease settings [5]. Early longitudinal analyses showed that the frequency of 1q21 gain increases from precursor plasma‐cell disorders to relapsed MM and is associated with disease progression [6]. More recent evaluations have reinforced the association between 1q abnormalities and high‐risk disease [7]. Increased 1q21 copy number and CKS1B expression have also been linked to inferior survival [8]. Approximately one‐third to two‐fifths of newly diagnosed patients harbor +1q, with a higher frequency observed in advanced or relapsed disease [9]. Importantly, gain(1q21), generally defined as three copies, and amp(1q21), defined as four or more copies, are not biologically equivalent, and increasing copy number is associated with progressively poorer outcomes [10]. Large contemporary cohorts further show that the prognostic effect of +1q varies according to clonal burden, treatment setting, and the accompanying cytogenetic profile [11].

The clinical behavior of +1q is strongly influenced by the genomic background in which it occurs. Coexisting chromosomal abnormalities can markedly worsen the prognosis associated with 1q21 abnormalities [12]. Patients with 1q21 gain treated with bortezomib‐based regimens may also have inferior outcomes, indicating that this lesion retains clinical relevance in the setting of proteasome‐inhibitor therapy [13]. Recognition of this heterogeneity contributed to the incorporation of 1q abnormalities into the Second Revision of the International Staging System (ISS) [14]. Conventional cytogenetic complexity provides another layer of risk, as complex karyotypes are independently associated with poor prognosis in MM [15]. These observations suggest that an apparently modest increase in 1q21 copy number may have a very different biological meaning when it occurs together with extensive chromosomal instability.

The genes affected by 1q21 copy number changes may help explain this variation in clinical behavior. Among the candidate genes in this region, interleukin‐6 receptor (IL6R) is of particular interest because interleukin‐6 (IL‐6) signaling is closely linked to plasma‐cell survival and treatment response. Functional studies have shown that increased IL6R expression in 1q21‐amplified MM cooperates with ADAR1‐P150 to enhance STAT3 signaling and oncogenic activity [16]. More recent clinical and experimental evidence has shown that 1q‐positive MM exhibits increased IL6R expression together with reduced CD38 expression, providing a mechanistic connection between 1q status, IL‐6/JAK/STAT signaling, and a treatment‐relevant plasma‐cell phenotype [17]. IL‐6 signaling has also been implicated in reduced sensitivity to bortezomib through STAT3/Notch signaling [18]. Work examining the bone marrow (BM) microenvironment further supports a role for the IL‐6/IL6R/JAK2/STAT3 pathway in myeloma‐cell survival, proliferation, and drug response [19]. These studies establish IL6R as a biologically relevant component of the 1q21 region, but gene dosage alone may not fully account for the marked differences in IL6R expression or clinical behavior among individual +1q cases.

Three‐dimensional genome organization provides a possible regulatory layer connecting structural genomic alterations with transcriptional output. Topologically associating domains (TADs) define local chromatin neighborhoods that influence enhancer‐promoter communication, and changes in domain boundaries can alter gene regulation [20]. In MM, high‐throughput chromosome conformation capture (Hi‐C) studies have demonstrated widespread spatial genome disorganization associated with copy number variation (CNV) [21]. Complex structural alterations in cancer can also reconfigure regulatory domains and generate enhancer‐hijacking events associated with oncogene activation [22]. The relationship between structural variation and three‐dimensional genome architecture is now recognized as an important mechanism of dysregulated gene expression in cancer [23]. A systems‐medicine analysis of chr1q‐amplified MM integrated genomic, epigenomic, transcriptomic, and three‐dimensional chromatin data and identified preserved regulatory domains and a PBX1–FOXM1 transcriptional program associated with high‐risk disease [24]. That study established that chr1q copy number changes can be interpreted in the context of three‐dimensional regulatory architecture, but the locus‐specific relationship between 1q21 gain, IL6R expression, and local chromatin organization remains poorly defined in clinically aggressive disease.

We report an elderly patient with newly diagnosed MM characterized by 1q21 gain, del(13q), a complex near‐triploid karyotype, rapid failure of bortezomib–dexamethasone induction, and exceptionally short survival. We integrated clinicopathologic assessment, fluorescence in situ hybridization (FISH), conventional karyotyping, whole‐genome sequencing (WGS), comparative transcriptomics, and Hi‐C to examine the genomic and regulatory context of this aggressive phenotype. 1q21 gain is accompanied by altered IL6R‐associated domain organization, increased enhancer‐promoter connectivity, and marked IL6R expression, offering a structural perspective on how 1q21 abnormalities may influence gene regulation beyond copy number change alone.

## 2 Case Presentation

### 2.1 Patient Information and Clinical Presentation

In April 2022, an 80‐year‐old man was admitted to the First People's Hospital of Chengdu City, China, with a 2‐month history of dizziness and fatigue, accompanied by intermittent cough, sputum production, chest discomfort, and decreased appetite. A previous evaluation at a local hospital had revealed severe anemia, thrombocytopenia, and marked hyperglobulinemia. On admission, the patient was cachectic and pale, with a Karnofsky Performance Status score of 65. Laboratory examination showed a red blood cell count of 1.48 × 10^12^/L, hemoglobin of 55 g/L, and platelet count of 39 × 10^9^/L. Serum total protein was 128.2 g/L, albumin was 21.1 g/L, and globulin was 107.1 g/L. The β_2_‐microglobulin level was 8.29 mg/L, serum calcium was 1.92 mmol/L, and uric acid was 506 μmol/L.

### 2.2 Clinical Findings and Diagnostic Assessment

Serum protein electrophoresis identified an M protein concentration of 79.59 g/L. Immunofixation showed a monoclonal band in the IgG lane and abnormal double‐clonal bands in the λ lane, consistent with an IgG‐λ monoclonal component with λ free light‐chain involvement. BM flow cytometry identified approximately 35.40% abnormal plasma cells expressing CD38, CD138, and CD56, with partial CD117 expression, absent CD19 expression, and cytoplasmic λ light‐chain restriction (Figure 1A). BM aspirate smears showed marked plasmacytosis, with plasmablasts and immature plasma cells accounting for 77% of nucleated cells (Figure 1B). Histopathological examination of the BM biopsy revealed diffuse infiltration by λ‐restricted plasma cells, occupying approximately 90% of the marrow, with mild reticulin fibrosis (Figure 1C,D). Chest computed tomography identified multifocal rib lesions with focal cortical destruction. Based on the clinical, laboratory, imaging, and pathological findings, the patient was diagnosed with IgG‐λ MM with λ free light‐chain involvement, classified as Durie–Salmon Stage IIIA and ISS Stage III.

**FIGURE 1:**
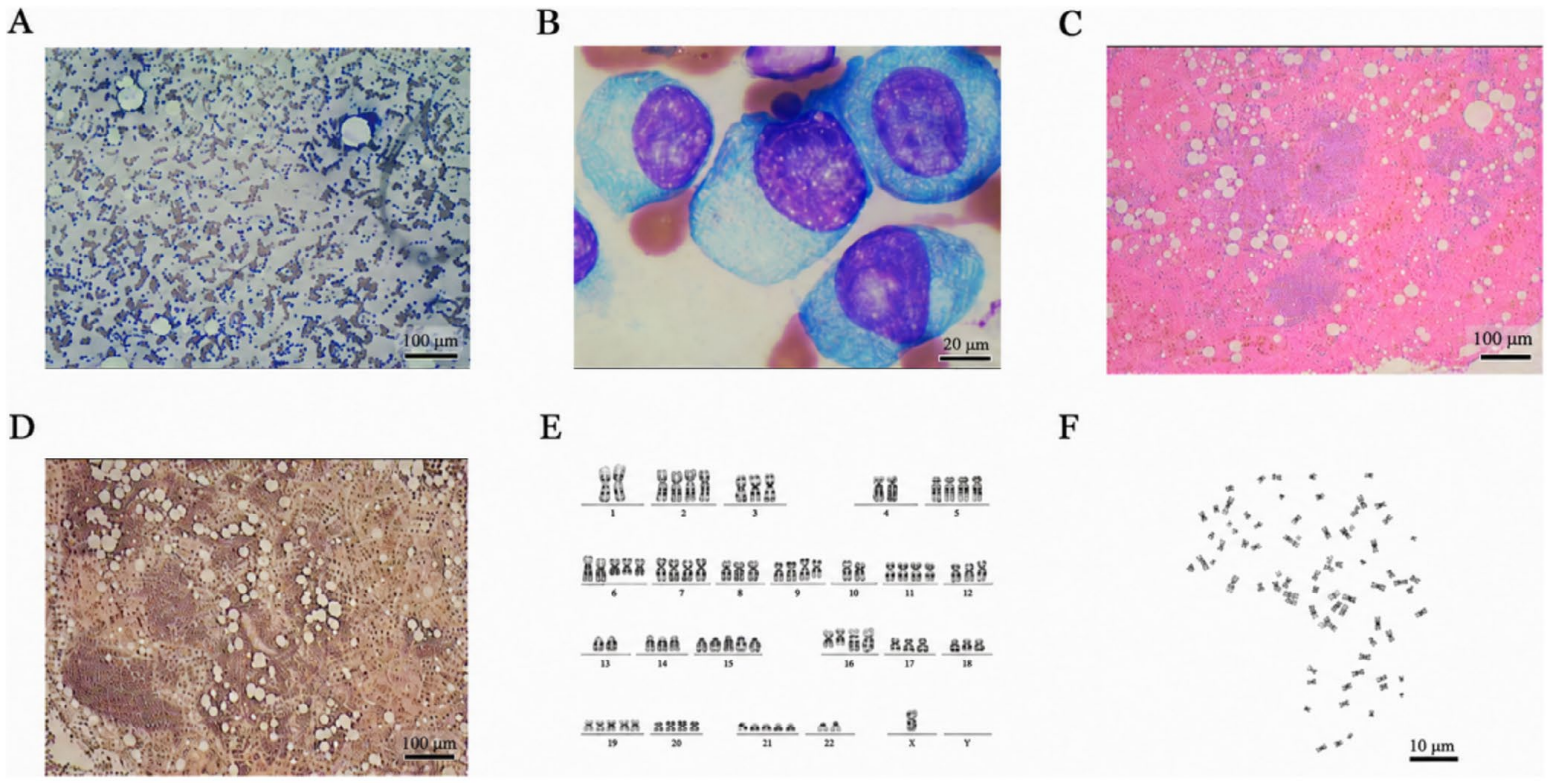
Baseline clinicopathologic characteristics of the patient. (A) Bone marrow flow cytometry identified an abnormal monoclonal plasma‐cell population. (B) Bone marrow smear showed marked plasmacytosis with immature morphology. (C) Bone marrow biopsy showed diffuse plasma‐cell infiltration. (D) Immunohistochemistry supported a lambda‐restricted plasma‐cell neoplasm. (E) G‐banded karyogram showed a complex near‐triploid karyotype. (F) Representative metaphase spread from conventional cytogenetic analysis.

### 2.3 Therapeutic Interventions and Follow‐Up

The patient underwent plasma exchange on April 29, 2022, followed by the first cycle of bortezomib plus dexamethasone in May 2022. Following treatment, hemoglobin increased to 88 g/L, whereas the platelet count remained low at 28 × 10^9^/L. Serum globulin initially decreased after plasma exchange but subsequently increased to 87 g/L by the end of the treatment cycle. On June 4, 2022, the patient was readmitted with persistent fatigue, nausea, poor appetite, and marked frailty. His body weight was 49 kg. Laboratory examination showed hemoglobin of 61 g/L, platelet count of 36 × 10^9^/L, serum globulin of 125.6 g/L, and β_2_‐microglobulin of 10.86 mg/L. In view of the persistent clinical symptoms and worsening laboratory findings, primary treatment resistance was considered. The patient underwent further plasma exchange, followed by ixazomib plus dexamethasone.

The patient was discharged on June 15, 2022. At discharge, hemoglobin was 73 g/L, platelet count was 69 × 10^9^/L, serum globulin was 99 g/L, and β_2_‐microglobulin was 10.39 mg/L. Although nausea had improved, poor appetite and marked frailty persisted. The discharge assessment represented the last documented in‐person clinical evaluation. No standardized quality‐of‐life assessment was recorded. Approximately 1 month after the final treatment, telephone follow‐up with the patient's family confirmed that he had died.

### 2.4 Cytogenetic and Genomic Characterization

Cytogenetic examination was performed on BM samples collected at diagnosis. Interphase FISH was conducted using probes targeting CKS1B (1q21), RB1 (13q), TP53 (17p), and recurrent IGH translocations, including t(4;14), t(11;14), t(14;16), and t(14;20). FISH identified 1q21 gain involving CKS1B and deletion of 13q involving RB1. Conventional G‐banded chromosome analysis revealed a complex near‐triploid karyotype (Figure 1E,F). The composite karyotype was 76–77(3n),X,‐X,‐Y,‐1,+2,‐4,+5,del(6)(q21),+del(6)(q21) × 2,+7,+9,‐10,+11,‐13,+15,+15,der(16)t(1;16)(q21,q22),+der(16)t(1;16)(q21,q22),+19,+19,+20,+21,+21,‐22(cp17)/46,XY(3).

WGS was performed on the diagnostic BM sample to characterize genomic copy number alterations. Regional copy number profiles were examined across chromosomes 1 and 13 using a detection window of 2042 bp. Within the 1q21 region, 5794 of 5827 genomic intervals exhibited copy number gain, comprising 4710 intervals with three copies and 1084 intervals with four copies. The remaining 33 intervals retained a copy number of 2. The gained region included IL6R and other genes located within 1q21 (Figure 2A). Chromosome 13q showed extensive copy number loss, with a single‐copy state predominating across the affected region (Figure 2B). The combined FISH, karyotyping, and WGS findings established the coexistence of 1q21 gain, 13q loss, and extensive chromosomal abnormalities in this patient.

**FIGURE 2:**
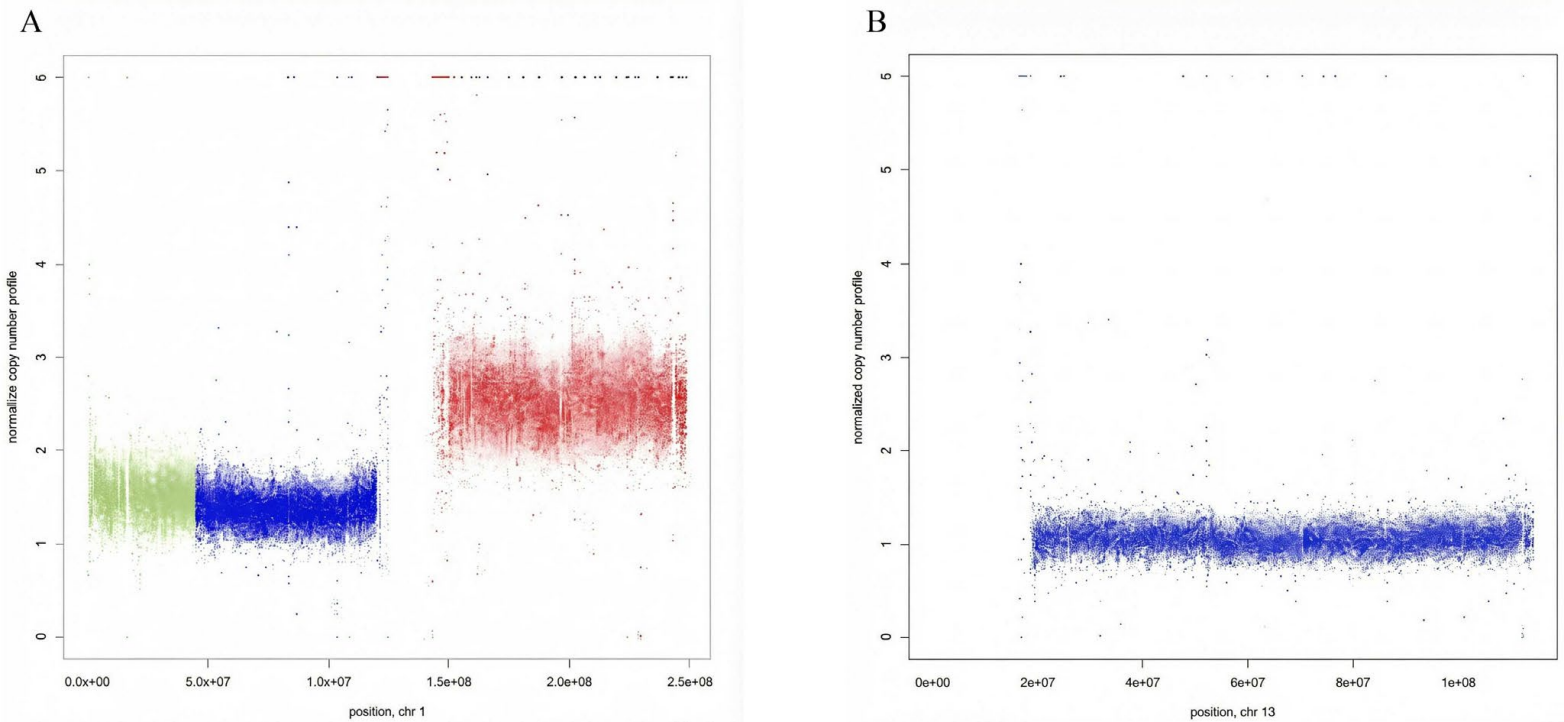
Copy number alterations involving chromosomes 1 and 13. (A) Genome‐wide copy number profile of chromosome 1 showing broad gain involving 1q21. (B) Copy number profile of chromosome 13 showing broad loss of 13q.

### 2.5 Transcriptomic Characterization

Comparative transcriptomic analysis was performed using the diagnostic BM sample and a pretreatment BM sample from a patient with MM without 1q21 gain. Both samples were processed using the same experimental workflow. Total RNA was extracted using TRIzol reagent. RNA concentration and purity were assessed using a NanoDrop 2000 spectrophotometer, and RNA integrity was evaluated using an Agilent 2100 Bioanalyzer. Messenger RNA was isolated by oligo(dT) magnetic bead enrichment, followed by fragmentation, first‐ and second‐strand cDNA synthesis, end repair, adapter ligation, and PCR amplification. Library quality was assessed before paired‐end sequencing (PE150) on the Illumina NovaSeq 6000 platform. Sequencing generated 6.8 Gb of data for the index sample and 6.2 Gb for the comparator sample.

Raw sequencing reads underwent quality filtering to remove adapter contamination and low‐quality sequences. Sequence alignment was performed against the human reference genome (hg19) using TopHat (v2.1.1), and transcript abundance was quantified using Cufflinks (v2.2.1). Gene expression differences between the two samples were evaluated based on fold changes (FCs). Functional enrichment analyses were conducted using Gene Ontology (GO) and Kyoto Encyclopedia of Genes and Genomes (KEGG) annotations, with the R package clusterProfiler and Benjamini–Hochberg adjustment for multiple testing.

Transcriptomic profiling revealed substantial differences in gene expression between the patient and comparator samples (Figure 3A). KEGG enrichment analysis identified pathways related to cancer signaling, phosphoinositide 3‐kinase–protein kinase B (PI3K–Akt) signaling, phospholipase D signaling, apelin signaling, platelet activation, viral protein interaction with cytokine and cytokine receptor, and hematopoietic cell lineage (Figure 3B). Examination of representative genes within or associated with the 1q21 region showed increased expression of IL6R, BCL9, CKS1B, and ILF2 in the index sample (Figure 3C,D). Among these genes, IL6R exhibited the greatest increase in expression, providing the basis for further examination of its local chromatin architecture.

**FIGURE 3:**
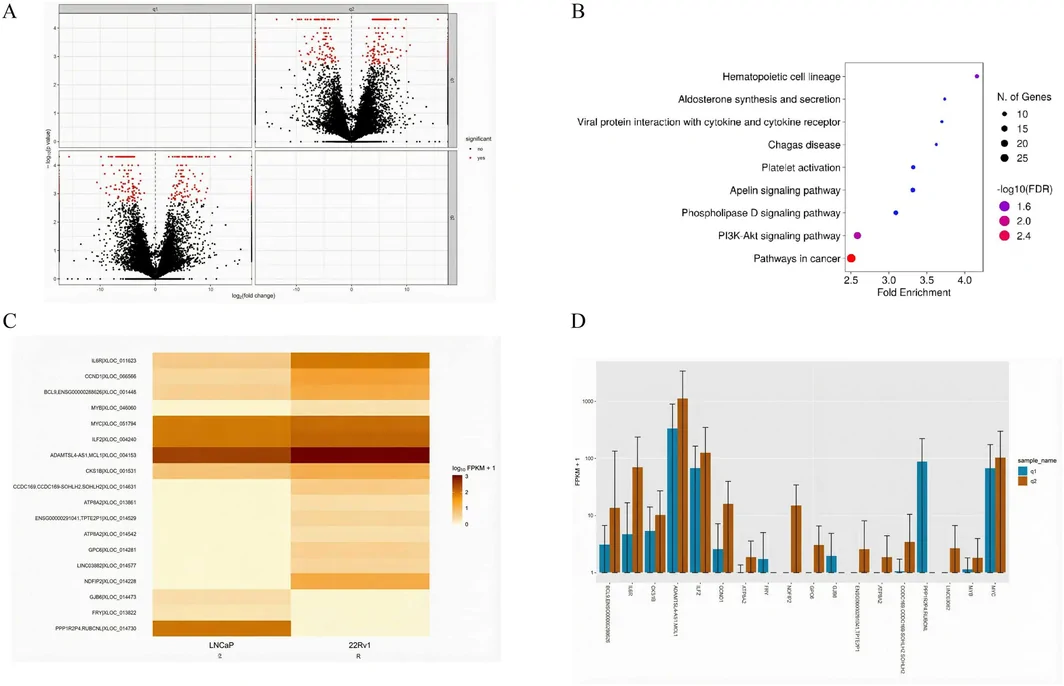
Transcriptomic alterations associated with 1q21 gain. (A) Differentially expressed genes between the control sample and the 1q21‐gain sample. (B) KEGG pathway enrichment of the dysregulated genes. (C) Heatmap of representative differentially expressed genes. (D) Expression abundance of selected genes in the control sample and the 1q21‐gain sample. q1, comparator MM sample without 1q21 gain; q2, index MM sample with 1q21 gain.

### 2.6 IL6R‐Associated Chromatin Organization

Hi‐C was performed on the diagnostic BM sample and the same comparator sample used for transcriptomic analysis. Fresh cells were cross‐linked with 2% formaldehyde for 10 min at room temperature, and the reaction was terminated using glycine at a final concentration of 0.125 M. Following cell lysis and nuclear isolation, chromatin was digested using the restriction endonuclease DpnII. Biotin‐labeled nucleotides were incorporated during end repair, followed by proximity ligation, reversal of cross‐links, and DNA purification. Purified DNA was fragmented to approximately 300–700 bp, and ligation products were enriched using streptavidin magnetic beads. Library concentration and fragment size were assessed using Qubit fluorometry and Agilent 2100 analysis, followed by quantitative PCR for library quantification. Libraries were sequenced on the Illumina NovaSeq 6000 platform using paired‐end 150‐bp reads.

Raw Hi‐C reads were filtered to remove adapter sequences, ambiguous bases, and low‐quality reads. Sequencing quality was evaluated using Q20, Q30, and GC content. Reads were aligned to the human reference genome using BWA‐MEM, and HiC‐Pro (v2.10.0) was used to distinguish valid interaction pairs from invalid ligation products and duplicated reads. Chromatin contact matrices were generated at multiple resolutions, with Knight‐Ruiz normalization applied for contact‐map analysis. TADs were identified using TadLib at 20‐kb resolution. Domain filtering incorporated the HOMER inclusion ratio (IR > 1), with domains shorter than five bins excluded. Local domain insulation and directionality were examined to characterize changes in chromatin organization. Chromatin loops were identified at 10‐kb resolution using a modified HICCUPS procedure with a false discovery rate threshold of 0.01. Promoter regions were defined as the 2‐kb intervals upstream of annotated transcription start sites (TSSs), while non‐promoter loop anchors were evaluated as candidate enhancer‐associated regions.

The analysis focused on the IL6R locus at chr1:154405193–154469450, encompassing three annotated IL6R transcripts. In the sample with 1q21 gain, all three transcripts were located within a single TAD, whereas the corresponding transcripts spanned two adjacent domains in the comparator sample. The 1q21‐gain sample showed expansion of the local regulatory domain, incorporating seven candidate enhancer elements into the same domain as IL6R. Increased enhancer‐promoter contacts were observed, accompanied by reduced local domain insulation and interactions involving regulatory regions outside the original domain.

## 3 Discussion

The most notable feature of this case was the convergence of an aggressive clinical course with a complex genomic and regulatory profile. The patient had FISH‐defined 1q21 gain together with del(13q) and a complex near‐triploid karyotype, extensive marrow involvement, severe cytopenias, and rapid treatment failure. WGS confirmed broad copy number alteration across 1q21, while transcriptomic analysis showed marked IL6R upregulation. Hi‐C further identified differences in local TAD organization and enhancer‐promoter contacts at the IL6R locus compared with the control sample. The value of this case is its distinctive feature: the patient‐level integration of cytogenetic abnormalities, copy number changes, gene expression, and locus‐specific three‐dimensional chromatin architecture in the same clinically aggressive 1q21‐positive MM case.

Clinical studies indicate that the prognostic effect of 1q21 abnormalities depends strongly on the cytogenetic and clinical context. Minguela et al. showed that the effect of 1q21 gain varies according to concurrent cytogenetic abnormalities and first‐line treatment, with del(13q) among the lesions associated with a less favorable clinical context [25]. Complex karyotype independently identifies patients with poor outcomes and reflects a high degree of chromosomal instability [15]. The inclusion of 1q abnormalities in the R2‐ISS also reflects the clinical relevance of +1q within a broader risk framework [14]. These observations are relevant to the present patient, in whom 1q21 gain occurred together with del(13q) and a highly complex near‐triploid karyotype. His rapid clinical deterioration cannot be attributed to 1q21 status alone. He was 80 years old, had a Karnofsky Performance Status score of 65, approximately 90% marrow tumor involvement, severe anemia and thrombocytopenia, and marked frailty. Each of these factors could have contributed to treatment failure and short survival. The clinical course should be interpreted as the result of multiple adverse features occurring simultaneously, with 1q21 gain representing one component of this high‐risk setting.

The IL6R findings should be considered in the same context. Teoh et al. showed that IL6R–STAT3–ADAR1 signaling promotes oncogenic activity in MM with 1q21 amplification [16], while Kuroki et al. reported increased IL6R expression and reduced CD38 expression in 1q‐positive MM [17]. Experimental work has also linked IL‐6/STAT3 signaling with reduced sensitivity to bortezomib [18]. Our observation of marked IL6R expression in a 1q21‐gain sample is consistent with this literature, but it does not establish that IL6R activation caused the patient's refractory disease. The patient differed from experimental models and cohort studies in several clinically important respects, particularly advanced age, severe frailty, high marrow tumor burden, del(13q), and extensive karyotypic abnormalities.

The three‐dimensional chromatin findings provide the main point of distinction from most previous clinical reports of 1q21‐positive MM. Wu et al. demonstrated that CNV in MM is associated with broad changes in three‐dimensional genome organization, including alterations in TAD boundaries and chromatin compartments [21]. More recently, Trasanidis et al. integrated genomic, epigenomic, transcriptomic, and three‐dimensional chromatin data in chr1q‐amplified MM and identified a PBX1–FOXM1 transcriptional program linked to preserved regulatory domains and reprogrammed super‐enhancers [24]. In cancer more broadly, structural alterations can change regulatory domains and create enhancer‐hijacking configurations associated with abnormal gene activation [22, 23]. Our case differs from these studies in its locus‐specific focus on IL6R in a patient with FISH‐defined 1q21 gain. The observed TAD reorganization, incorporation of distal enhancers into the local regulatory domain, and increased enhancer‐promoter contacts occurred together with marked IL6R expression. These observations identify IL6R as a candidate locus for further study in 1q‐positive MM and suggest that copy number status may coexist with changes in local regulatory architecture. They do not demonstrate that 1q21 gain produced the chromatin changes or that these changes caused IL6R overexpression.

There are several limitations. First, this is a single case, and both the transcriptomic and Hi‐C comparisons rely on one patient sample and one comparator. Interindividual variation cannot therefore be separated from disease‐associated variation, and the findings cannot be generalized to other patients with 1q21 gain. Second, no functional perturbation experiments were performed. The study cannot determine whether altered TAD organization or enhancer‐promoter contacts are required for IL6R expression, nor whether changing these regulatory elements would affect IL6R signaling, myeloma‐cell survival, or drug sensitivity. Third, Hi‐C contact frequencies can themselves be influenced by CNV, an important consideration when interpreting contact differences within a copy number‐gained region [21]. Orthogonal validation using independent samples and locus‐focused chromatin assays will be required to establish whether the observed IL6R architecture is reproducible. Finally, the patient's short survival cannot be assigned to IL6R dysregulation or 1q21 gain because age, frailty, tumor burden, severe cytopenias, del(13q), and complex karyotype provide several competing explanations for the clinical outcome.

1q21 abnormalities should not be interpreted solely by copy number or as isolated cytogenetic events. Their clinical meaning may depend on the accompanying genomic background and, potentially, on the regulatory state of genes within the affected region. In this patient, integrated genomic profiling identified an association among 1q21 copy number gain, marked IL6R expression, and altered local chromatin organization. These findings are hypothesis‐generating and justify testing the IL6R locus in larger independent cohorts of 1q‐positive MM using matched genomic, transcriptomic, and chromatin‐conformation data. Functional perturbation of candidate enhancers and IL6R regulatory elements will be necessary before any causal relationship with disease aggressiveness or treatment response can be established.

## 4 Conclusion

This case illustrates that the clinical significance of 1q21 gain should be interpreted together with concurrent cytogenetic abnormalities, particularly del(13q) and complex karyotype, rather than on copy number alone. Integrated genomic analyses identified broad 1q21 copy number gain, marked IL6R upregulation, and altered local chromatin organization at the IL6R locus, although these findings represent associations and do not establish causality. From a clinical perspective, comprehensive cytogenetic and genomic assessment may help refine risk interpretation in patients with 1q21‐positive MM whose clinical course appears disproportionate to copy number status alone. Larger independent cohorts and functional studies are required before IL6R‐associated chromatin features can be considered for prognostic stratification or therapeutic decision‐making.

## Author Contributions

**Haibo Liu:** conceptualization, methodology, resources, writing – original draft. **Mengshi Chen:** methodology. **Yue Wang:** writing – original draft. **Ming Chen:** writing – original draft. **Dong He:** writing – review and editing. **Yanju Li:** formal analysis, investigation. **Kaiji Zhang:** conceptualization, writing – review and editing, supervision, investigation, writing – original draft.

## Funding

This work was supported by funding from the Science and Technology Department of Sichuan Province (No. 2020YJ0438), National Health Commission Capacity Building and Continuing Education Center (NHC Party School), the 2025 Chronic Disease Management Research Project (No. GWJJMB202510010017).

## Ethics Statement

This study was approved by the Ethics Committee of the First People's Hospital of Chengdu City, Chengdu, China (Approval No. ZXKY No. 002, 2020).

## Consent

Written informed consent for participation was obtained from the patient's next of kin.

## Conflicts of Interest

The authors declare no conflicts of interest.

## Data Availability

The datasets generated and/or analyzed during the current study are available in the Genome Sequence Archive (GSA) of the National Genomics Data Center (NGDC), China National Center for Bioinformation, under accession number HRA007587.
